# Mirizzi syndrome type IV associated with cholecystocolic fistula: a very rare condition- report of a case

**DOI:** 10.1186/1471-2482-7-6

**Published:** 2007-05-27

**Authors:** George Chatzoulis, Andreas Kaltsas, Lazaros Danilidis, John Dimitriou, Ioannis Pachiadakis

**Affiliations:** 1 Department of Surgery, 424 Military Hospital Thessaloniki, Greece; 2 Department of Surgery, St Loukas Hospital Thessaloniki, Greece; 3 Department of Gastroenterology, 424 Military Hospital Thessaloniki, Greece

## Abstract

**Background:**

Mirizzi syndrome is a rare complication of prolonged cholelithiasis with presence of large, impacted gallstone into the Hartman's pouch, causing chronic extrinsic compression of common bile duct (CBD). Fistula formation between the CBD and the gallbladder may represent an outcome of that condition. According to Mirizzi's classification and Csendes's subclassification, Mirizzi syndrome type IV represents the most uncommon type (4%).

Spontaneous biliary-enteric fistulas have also been rarely reported (1.2–5%) in a large series of cholecystectomies. Cholecystocolic fistula is the most infrequent biliary enteric fistula, causing significant morbidity and representing a diagnostic challenge.

**Case presentation:**

We describe a very rare, to our knowledge, combination of Mirizzi syndrome type IV and cholecystocolic fistula. A 52 year old male, presented to our clinic complaining of episodic diarrhea (monthly episodes lasting 16 days), high temperature (38°C–39°C), right upper quadrant pain without jaundice. The definitive diagnosis was made intraoperatively. Magnetic Resonance Imaging (MRI) and Endoscopic Retrograde Cholangiopancreatography (ERCP) demonstrated the presence of Mirizzi syndrome with cholecystocolic fistula formation. The patient was operated upon, and cholecystectomy, cholecystocolic fistula excision and Roux-en-Y biliary-enteric anastomosis were undertaken with excellent post-operative course.

**Conclusion:**

Appropriate biliary tree imaging with ERCP and MRI/MRCP is essential for the diagnosis of Mirizzi syndrome and its complications. Cholecystectomy, fistula excision and biliary-enteric anastomosis with Roux-en-Y loop appears to be the most appropriate surgical intervention in order to avoid damage to Calot's triangle anatomic elements. Particularly in our case, ERCP was a valuable diagnostic tool that Mirizzi syndrome type IV and cholecystocolic fistula.

## Background

Mirizzi syndrome (MS) in association with biliary-enteric fistula is an extremely rare combination. To our knowledge there is only one report that refers to the rare coexistence of Mirizzi syndrome Type II with a parapapillary choledochoduodenal fistula [1].

The definitive diagnosis for these two rare internal fistulas was reached preoperatively, by ERCP. It is important to identify Mirizzi syndrome and fistula formation because of the serious morbidity and mortality related to the condition [2-4]. Treatment choice is equally important as the chronic biliary tree inflammation and subsequent bile ducts anatomic alterations necessitate a meticulous surgical approach. We present here a very rare combination of Mirizzi syndrome type IV and cholecystocolic fistulas, diagnostic approach and surgical therapy.

## Case presentation

**Figure 1:**
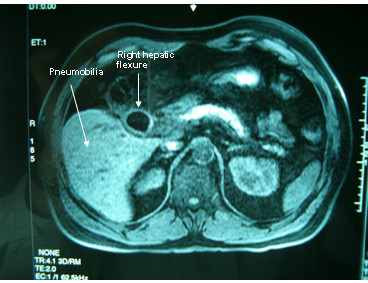
Spiral CT with evidence of pneumobilia and suspicion of cholecystocolic fistula.

**Figure 2:**
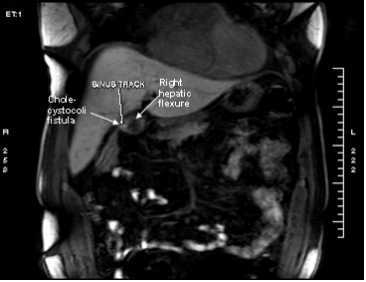
MRI:T1 and T2 weighed images with iv contrast Gadolinium- Bopta, revealing fistulous tract between the right colonic flexure and gallbladder (cholecystocolic fistula) and a large gallstone (2 cm).

**Figure 3:**
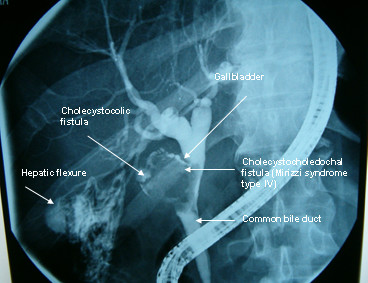
Endoscopic retrograde cholangiopancreatography. Severe common bile duct stenosis, with wide cholecystocholedochal communication. A shrunk gallbladder with a large gallstone and a secondary formation of a fistoulous tract, the right hepatic flexure with the contrasted colonic haustration are the highlights of this image modality.

A 52 years old Caucasian male, presented himself to our clinic complaining of episodic diarrhea, fatigue, intermittent high fever and right upper quadrant pain. Nevertheless he was anicteric. Blood tests showed SGOT 89 U/L (normal 5–40 U/L), SGPT 115 U/L(normal 5–40 U/L), Alkaline Phosphatase 153 U/L(normal < 106 U/L), γGT 107 U/L(normal < 50 U/L) with normal bilirubin levels 0.5 mg/dl (normal values for total bilirubin 0,2-1 mg/dl). Afterwards U/S examination revealed pneumobilia and large gallstone impacted in a shrunk gallbladder and a narrow, ill-defined common bile duct. Subsequently, CT scan revealed the large gallstone and pneumobilia (Fig. 1). Finally, MRI and MRCP indicated the presence of a sinus tract between the gallbladder fossa and the hepatic flexure, and a possible cholecystobiliary fistula (Fig. 2). The patient underwent colonoscopy in order to investigate the possibility of inflammatory bowel disease (Crohn's disease) being the underlying condition. The endoscopy revealed a small area in the hepatic flexure with mucosal inflammation but no fistulous tract opening. Consecutively, ERCP undertaken, showed Mirizzi syndrome type IV, fistulous tract formation, a large impacted gallstone and severe stricture of the common bile duct, with a simultaneous revealing of the cholecystocolic fistula delineating the colonic haustration (Fig.3).

The definitive diagnosis of these two internal fistulas was reached intraoperatively. Right subcostal incision with left and midline extension was performed and two severe anatomic alterations were observed. The first was the cholecystocolic communication with the right hepatic flexure and the second was the shrunken gallbladder with the associated presence of a wide communication (about 3 cm), between the gallbladder and common bile duct. After excision of the cholecystocolic tract and suturing in two layers of the colonic wall, the extraction of a large stone (3 cm in diameter) through an incision of the gallbladder fundum and after two consecutive intraoperative cholangiograms the definition of Mirizzi syndrome type IV was more than obvious. The final therapy was partial cholecystectomy and side- to- side choledochojejunostomy Roux en Y.

No malignancy was observed in any section of the cholecystectomy specimen [5].

The postoperative course was uneventful. The patient was discharged a week after the operation.

## Discussion and conclusion

Mirizzi syndrome is a rare complication (frequency about 1%) of chronic cholecystitis and prolonged cholelithiasis, which consists of inflammatory process of gallbladder wall and direct compression (Mirizzi syndrome type I) or erosion (Mirizzi syndrome type II, III, IV) of the common bile duct and subsequent fistulous formation [6].

**Figure 4:**
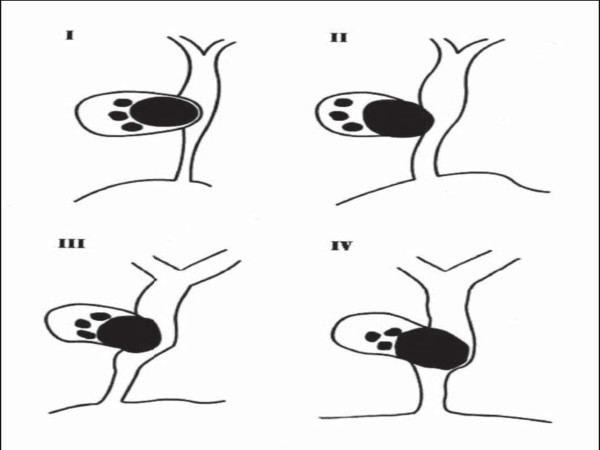
Schematic representation of Csendes classification for Mirizzi syndrome [4].

After Mirizzi and Mc Sherry [7] classification, the most recent and predominant classification was made by Csendes et al [8]. According to it, Mirizzi syndrome is divided in four types depending on the size of the destruction of the common bile duct (Fig. 4)[4]. A wide communication that included the entire circumference of the CBD (Over 66% of the common bile duct diameter) classifies this case as Mirizzi syndrome type IV [9].

In a large study (219 patients), Csendes et al [8], reported that 11% of their patients with Mirizzi syndrome had type I lesions, 41% had type II lesions, 44% had type III lesions, and 4% had type IV lesions.

Clinical manifestation of this syndrome includes recurrent cholangitis, jaundice, right upper quadrant pain, and abnormal liver biochemistry [10,11]. It is remarkable that this patient was anicteric.

The gold standard in diagnosis of Mirizzi syndrome is ERCP [12]. In this case ERCP was a valuable diagnostic tool that revealed the bile duct stenosis, the impacted gallstone, the wide cholecystocholedochal fistula, and the colonic haustration of the cholecystocolic fistula. Another helpful diagnostic method in this case was Magnetic Resonance Cholangiopancreatography (MRCP). In this case MRCP demonstrated neither the fistulas nor the choledochal stenosis, but it raised a suspicion of an abnormal communication between the gallbladder and the common bile duct. According to the literature, MRCP usually is a sensitive diagnostic tool for the detection of fistulas [13].

Mirizzi syndrome is a rare complication of cholelithiasis that represents a dangerous alteration of the anatomy and bears the potential to lead to significant morbidity and biliary injury, particularly in the laparoscopic era. In this condition the cystic duct usually runs parallel to the CBD (10% of cholangiograms) [14]. Preoperative recognition of this variation is important to avoid inadvertent ligation or severance of the bile duct. An attempt to expose Calot's triangle may lead to severe bile duct injury such as: i) iatrogenic communication between the gallbladder and CBD [4], ii) complete transection of CBD after dissection of the gallbladder neck [2,12,15], iii) tear of CBD [2]. Additionally, a high coincidence of Mirizzi syndrome and gallbladder cancer has been reported in several studies[16-18].

The intraoperative confirmation of Mirizzi syndrome was made after the extraction of the large gallstone. The contracted gallbladder, the inflammatory wall and the subsequent adherence with the common bile duct (CBD) resulted to the wide communication (fistulas diameter: = 3.5 cm) [4]. After temporary stenting of the CBD through the fistula opening we classified the condition as Mirizzi syndrome type IV which represents the most uncommon type [8].

The cholecystoenteric fistula usually develops insidiously, and an association with gallstones is always present. Cholecystoenteric fistula appears in 1.2 to 5% of patients with acute and chronic cholecystitis in a large series of cholecystectomies [19,20]. About 50% of diagnosis for biliary-enteric fistulas is preoperative. ERCP is the most important diagnostic tool in order to identify the presence of biliary-intestinal fistula. Two other significant diagnostic methods MRI and MRCP set up a high index of suspicion revealing the fistulous tract [21]. Subsequently ERCP was almost pathognomic as it demonstrated the colonic haustration. ERCP, barium enema, colonoscopy with contrast fistulography, technetium 99 m scintiscan, and morphine augmented hepatobiliary cholescintigraphy have been proved to be efficacious in the detection of the cholecystocolic fistula [3,22-25]. In this case, colonoscopy was unable to detect the fistulous tract, but the notification of the inflammatory area of the hepatic flexure was very helpful in the overall diagnosis. A causative correlation between Crohn's disease and cholecystocolic fistula formation was also precluded in this case, by colonoscopy [26,27].

The most common type of biliary-enteric fistula is cholecystoduodenal (75%); cholecystocolic is next common (10–20%), with a variety of other types being less frequent (15%) [28-30].

Pneumobilia, atrophic gallbladder with inflamed wall and impacted gallstone as U/S and CT findings, malabsortion with monthly diarrhea as the only clinical manifestation, were the main initial characteristics of this patient [21]. The potential causative agent of this fistula was mechanical erosion by the gallstone between the gallbladder infundibulum and the adjacent colon [31]. Chronic diarrhea, massive lower gastrointestinal hemorrhage, and gallstone ileus are the main severe complications of a cholecystocolic fistula [3,22,31-34]. The fistula's excision and repair of the hepatic flexure with primary closure was finally performed without any further postoperative complications.

The relatively high rate of inadvertent bile duct injury, as previously mentioned, reiterates the danger of Mirizzi syndrome. Laparoscopic cholecystectomy in MS type IV is hazardous and maintains a high threshold to open surgery. ERCP and intraoperative cholangiograms are significant diagnostic tools for MS. The proper surgical treatment of this fistula is partial cholecystectomy and side to side Roux-en-Y choledochojejunostomy. The inflammation and the high risk of fibrosis and stenosis make the primary repair of the fistula hazardous with increased morbidity and mortality rates, indicating Roux-en-Y hepaticojejunostomy the treatment of choice in Type IV Mirizzi syndrome [8,35]. On the other hand cholecystocolic fistula can be diagnosed and even treated by ERCP, and other methods as previously mentioned. Early operation with excision of the fistula and primary closure of the large bowel's defect is the treatment of choice[21,22,33]. Successful laparoscopic repair of isolated cholecystocolic fistulas has also been reported [21].

The rarity of this combination and the precocious recognition remains a challenge for the surgeon. ERCP remains the most powerful diagnostic tool and particularly in this case report approached with high accuracy the correct diagnosis. Primary repair of cholecystocolic fistula and minimal surgical maneuvers of Calot's triangle at the time of the operation for Mirizzi syndrome type IV in order to avoid bile duct injury are two important key points for successful treatment, of these clinical entities.

## Competing interests

The author(s) declare that they have no competing interests.

## Authors' contributions

All the authors have been involved in literature search, writing and final reviewing of this manuscript.

## Pre-publication history

The pre-publication history for this paper can be accessed here:


